# Stapling of an endobronchial suction tube with the bronchus during robot-assisted right lower lobectomy: a case report

**DOI:** 10.1186/s40792-021-01278-5

**Published:** 2021-08-23

**Authors:** Hiroto Tanaka, Teruhiro Aoki, Makoto Oda, Yoshimasa Inoue

**Affiliations:** 1 Department of Thoracic Surgery, Saiseikai Yokohama-shi Tobu Hospital, 3-6-1 Shimosueyoshi, Turumi Ward, Yokohama, Kanagawa 230-0012 Japan; 2 Department of Thoracic Surgery, Shin-yurigaoka General Hospital, 255, Aza-tsuko, Furusawa, Aso Ward, Kawasaki, Kanagawa Japan

**Keywords:** Intraoperative complication, Robot-assisted thoracic surgery, Anatomical lung resection

## Abstract

**Background:**

Troubleshooting intraoperative complications requires careful management, and the safest technique should be chosen. We recently experienced a unique intraoperative bronchial complication during pulmonary lobectomy in robot-assisted thoracic surgery (RATS). There is no consensus on whether to continue RATS or convert to a more familiar technique, such as video-assisted thoracic surgery (VATS) or thoracotomy, for intraoperative complications that occur during RATS, and the decision should be determined individually.

**Case presentation:**

A 74-year-old woman with primary lung adenocarcinoma (clinical stage IA2) underwent robot-assisted right lower lobectomy under one-lung ventilation and CO_2_ insufflation. Intraoperatively, the anesthesiologist placed the endobronchial suction tube in the right bronchus with intention of maintaining the right lung collapse, which was simultaneously stapled with the right lower bronchus during the right lower lobe bronchial closure using a robotic stapler. During robot-assisted manipulation, we removed the staples involved with the suction tube, one by one, using robotic-arm forceps and sutured the partially opened stump. Subsequently, the bronchial stump was covered with a pedicled pericardial fat pad. The postoperative course was uneventful, and the patient developed no complications when followed up 8 months after discharge. Hence, we could rectify this intraoperative bronchial complication using a robot-assisted technique and avoid conversion to VATS or thoracotomy.

**Conclusion:**

The precise manipulation techniques in RATS contributed to facilitate the successful execution of surgical procedures, such as staple removal and re-suturing of the bronchial stump and may be a useful as a method for such troubleshooting such intraoperative complications.

**Supplementary Information:**

The online version contains supplementary material available at 10.1186/s40792-021-01278-5.

## Background

Robot-assisted thoracic surgery (RATS) has gained popularity in the field of general thoracic surgery as a novel, minimally invasive, surgical technique, since Melfi et al. [1] reported robot-assisted pulmonary resection in 2002. Compared with video-assisted thoracic surgery (VATS), RATS facilitates improved visibility with a three-dimensional stereoscopic view and magnified field of view; similarly, it allows for greater precision owing to multi-joint instrumentation and reduction in human hand tremors [2, 3]. Despite its advantages, RATS continues to encounter challenges, such as the increased preparation time, high cost, lack of tactile sensation during manipulation of the forceps [4, 5], and poor documentation on the perioperative complications. In this case report, we describe how a unique intraoperative bronchial complication, involving a suction tube during bronchial closure with a robotic stapler, could be rectified by procedures performed using a RATS technique.

## Case presentation

**Fig. 1:**
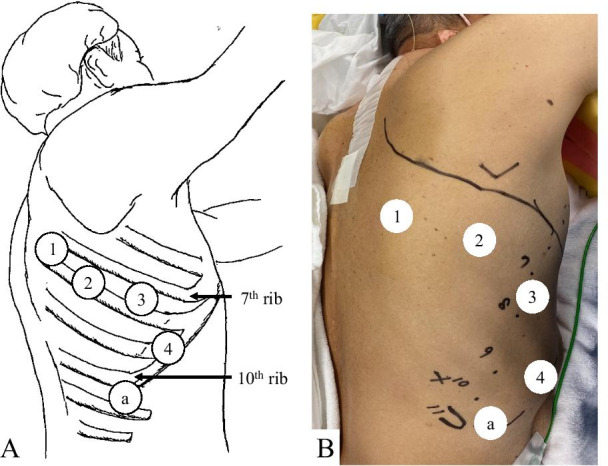
**A** Schema of the port placement in the right chest. Ports 1–4 are for the robotic arm. The other part (a) is for the assistants. **B** Preoperative design of port placement in the right chest. The line over the ports 1–3 is the incision performed in the case of emergency open thoracotomy. The patient’s head is at the top of the picture and the anterior chest is to the right of the picture

A 74-year-old woman (height, 148 cm; weight, 50 kg) was diagnosed with lung adenocarcinoma in the right lower lobe (RLL). The clinical stage was 1A2 (T1bN0M0, UICC 8th), and a robot-assisted right lower lobectomy with lymph node dissection was performed under one-lung ventilation with a double-lumen endobronchial tube. Port placement was performed according to the methods reported by Dylewski et al. [6]. We used the da Vinci surgical system type Xi (Intuitive Surgical, Sunnyvale, CA, USA). Three da Vinci arm ports using an 8-mm reusable metal da Vinci trocar were set in the seventh intercostal space: one below the subscapularis angle, the other on the posterior axillary line, and the third on the anterior axillary line; they were docked with a cadiere forceps, 0-degree lens scope, and curved bipolar dissector, respectively. A 12-mm metal port was placed on the ventral side of the eighth intercostal space, and this port was docked with tip-up fenestrated grasper and the da Vinci-equipped stapler. A 12-mm AIRSEAL access trocar (Conmed, Largo, FL, USA) was placed in the 10th intercostal space as CO_2_ insufflation combined assistant port, and the console operation was started (Fig. 1).

**Fig. 2:**
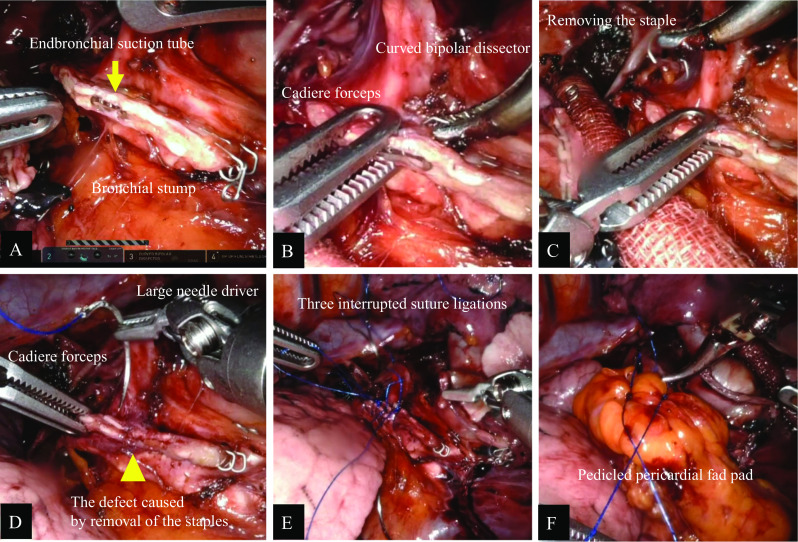
Intraoperative views. **A** Endobronchial suction tube involving the dissected right lower lobe bronchus (arrow). **B** Curved bipolar dissector allowed the surgeon to pinch the staple individually while grasping the bronchus with cadiere forceps to prevent it from moving. **C** All the staples involved with the suction tube were successfully removed. **D** Primary closure of the defect in the bronchial stump caused by the removal of the staple (arrow head). **E** Three interrupted suture ligations were placed at the defect in the bronchial stump. **F** Covering the bronchial stump with a pedicled pericardial fat pad

The surgery proceeded uneventfully before the bronchial resection. After dissecting the RLL bronchus by the da Vinci-equipped stapler, we found that an endobronchial suction tube (EST) had been pinched in the bronchial stump (Fig. 2a). There were no warnings, such as high resistance during clamping or stapling. The EST was placed to maintain the collapse of the right lung at the discretion of the anesthesiologist. Since the EST was firmly trapped in the staple line, the EST could not be withdrawn manually. Although we considered modifying our technique to VATS, which is a familiar approach, we attempted to see if the repair could be performed using RATS, with sufficient attention to safety (Additional file 1: Video). First, the bronchial stump was grasped with cadiere forceps and staples were grasped one by one with a curved bipolar dissector while observing the site under magnification to avoid unnecessary bronchial injuries (Fig. 2b). All staples involving the EST were extracted successively, and the EST was removed from the RLL bronchus (Fig. 2c, d). Next, we sutured the partially opened bronchial stump with three interrupted suture ligations using 3–0 Prolene (Fig. 2e) and covered the bronchial stump with a pedicled pericardial fat pad (Fig. 2f). No endobronchial defect was seen on the suture line on bronchoscopic evaluation, and no significant air leak was detected during a leakage test of the operative lung at a positive pressure of 20 cmH_2_O. A chest tube was inserted to check postoperative bleeding and air leakage. The operation was finished without any other adverse events, and the patient was transferred to the intensive care unit for close observation. The operation time was 3 h and 33 min, and the surgeon console time was 2 h and 45 min; intraoperative blood loss was minimal. No air leakage was found through the chest tube. The patient was discharged on the 5th postoperative day without complications. Furthermore, the patient experienced no perioperative complications during a follow-up of 8 months after discharge.

## Discussion

Upon searching PubMed and Google Scholar, we found two cases in which foreign bodies, such as plastic tubes, were involved with the staple line during pulmonary lobectomy [7, 8]. In both cases, the bronchial brokers were pinched with the bronchial stump, and removing the staple and re-suturing were performed manually.

A further literature search was conducted to find out how to deal with intraoperative bronchial problems. Previous reports have described a 0.1–1.5% occurrence of intraoperative bronchial injuries (Table 1) [9–12]. These injuries were caused by surgical manipulation or stapling of the bronchial stump. Regarding the repair of intraoperative bronchial injuries by VATS lobectomy, Flores et al. [9] reported that 12 of 633 cases had some injuries requiring additional non-elective procedures; these included one case of membranous injury of the bronchus that was repaired by conversion to thoracotomy. Decaluwe et al. [10] reported that among 3076 VATS patients, serious injuries occurred in 46 patients (1.5%), of which three (0.1%) were bronchial injuries. One of the three injuries was caused by surgical manipulation, another by stump injury with stapling, and the cause of the third was unknown. In all three cases, the injured bronchus was repaired by conversion to thoracotomy. Regarding intraoperative injuries during RATS, Cao et al. [11] reported that 35 of 1264 patients had injuries that required additional unscheduled procedures and included four bronchial injuries, three of which required thoracotomy to repair the injury and one required postoperative reoperation for repair. Ueno et al. [12] reported that intraoperative injuries occurred in eight (4.1%) of 192 cases of RATS (156 lobectomies and 36 segmentectomies) for primary lung cancer, including three (1.5%) bronchial injuries. In two cases, the bronchial wall near the stapled bronchial stump was injured, and in one case, bronchial injury occurred during the procedure of dissecting the peribronchial tissue. In all cases the repair was performed by primary closure with interrupted pledgeted suturing, and the bronchial stump was subsequently covered using a pedicled pericardial fad pad without conversion to VATS or thoracotomy. In summary, in cases of intraoperative bronchial injury in VATS, all patients underwent conversion to thoracotomy for repair. In contrast, in all RATS cases, repair was possible without conversion to thoracotomy. It is presumed that features, such as three-dimensional stereoscopic vision, magnification, and reduction of the tremors of the surgeon’s hand, improved the operative technique, and conversion to thoracotomy could be prevented. As shown in our surgical video (Additional file 1), although the Staples were deeply embedded in the bronchial wall, but the staple could be removed by grasping and fixing the bronchial stump with cadiere forceps and then using the strong grasping force of the curved bipolar dissector. If the Staple had been attached in the opposite direction, it would have been difficult to make surgical field and the staples could not have been removed in RATS.

**Table 1:** Previously reported cases of intraoperative complications during video- and robot-assisted pulmonary resection

Author (year)	Type of surgery	Evaluation period	Institution	Number of patients	Intraoperative major complication*	Bronchial injury
Flores (2011) [9]	VATS	2002–2010	Single	633	12 (2%)	1 (0.2%)
Decaluwe (2015) [10]	VATS	NA	Multi	3076	46 (1.5%)	3 (0.1%)
Cao (2019) [11]	RATS	2002–2018	Single	1264	35 (1.9%)	4 (0.3%)
Ueno (2020) [12]	RATS	2017–2019	Single	196	8 (4.2%)	3 (1.5%)

*VATS* video-assisted thoracic surgery, *RATS* robot-assisted thoracic surgery, *NA* not available

*Intraoperative major complications are defined as events that resulted in an additional unplanned major surgical procedure other than the planned lobectomy

According to previous reports [13, 14], intraoperative endobronchial suctioning that caused our intraoperative complication does not promote lung collapse. Previous studies reported that two mechanisms are mainly involved in the determination of rate of collapse of the nonventilated lung. The first mechanism is passive exhalation due to the inherent elastic recoil of the lung, which is usually over within 60 s of lung isolation. The second mechanism of lung collapse during one-lung ventilation is the uptake of remaining lung gases, and pulmonary collapse depends on changes in the rate of gaseous uptake in the alveoli. Therefore, any means to further deflate such as suctioning of the airway failed due to small airway closure. For this reason, intraoperative continuous endobronchial suctioning, as performed in this case, did not actually contribute to the collapse of the unventilated lung and was considered unnecessary. Furthermore, CO_2_ insufflation in the thoracic cavity allowed the surgical manipulation field to expand sufficiently in RATS. In the future, at our institution, if non-ventilated lung is insufficiently collapse intraoperatively, we should check up the position of the intubation tube is instead of suctioning the bronchus and wait for the collapse to progress over time. In addition, it is obvious that the surgeon should routinely confirm with the anesthesiologist before critical procedures as stapling the bronchus; therefore, the outcome, in this case, resulted from insufficient cooperation between the surgeon and anesthesiologist.

## Conclusions

RATS has shown great promise in performing precise surgical procedures that were previously difficult. In the present case, accurate RATS techniques facilitated staple removal and re-suturing of the bronchial stump and may be a beneficial method for such intraoperative complications.

## Supplementary Information

**Additional file 1.** The Staples were deeply embedded in the bronchial wall, but could be removed by using a curved bipolar dissector with strong grasping force while grasping and securing the bronchial stump with cadiere forceps.

## Data Availability

No applicable.

## Abbreviations

EST: Endobronchial suction tube
RATS: Robot-assisted thoracic surgery
RLL: Right lower lobe
VATS: Video-assisted thoracic surgery

## References

[1] Melfi FM, Menconi GF, Mariani AM, Angeletti CA (2002). Early experience with robotic technology for thoracoscopic surgery. Eur J Cardiothorac Surg.

[2] Cerfolio RJ, Bryant AS, Skylizard L, Minnich DJ (2011). Initial consecutive experience of completely portal robotic pulmonary resection with 4 arms. J Thorac Cardiovasc Surg.

[3] Cao C, Manganas C, Ang SC, Yan TD (2012). A systematic review and meta-analysis on pulmonary resections by robotic video-assisted thoracic surgery. Ann Cardiothorac Surg.

[4] Cerfolio RJ (2014). Total port approach for robotic lobectomy. Thorac Surg Clin.

[5] Paul S, Jalbert J, Isaacs AJ, Altorki NK, Isom OW, Sedarakyan A (2014). Comparative effectiveness of robotic-assisted vs thoracoscopic lobectomy. Chest.

[6] Dylewski MR, Ohaeto AC, Pereira JF (2011). Pulmonary resection using a total endoscopic robotic video-assisted approach. Semin Thorac Cardiovasc Surg.

[7] Soto RG, Oleszak SP (2006). Resection of the Arndt bronchial blocker during stapler resection of the left lower lobe. J Cardiothorac Vasc Aneth.

[8] Lee YH, Yang HM, Kim HC, Bahk JH, Seo JH (2015). Transection of a Coopdech bronchial blocker tip during bronchial resection for right upper lobectomy: a case report. Korean J Anesthesiol.

[9] Flores RM, Ihekweazu U, Dycoco J, Rizk NP, Rusch VW, Bains MS (2011). Video-assisted thoracoscopic surgery (VATS) lobectomy: catastrophic intraoperative complications. J Thorac Cardiovasc Surg.

[10] Decaluwe H, Petersen RH, Hansen H, Piwkowski C, Augustin F, Brunelli A (2015). Major intraoperative complications during video-assisted thoracoscopic anatomical lung resections: an intension-to-treat analysis. Eur J Cardiothorac Surg.

[11] Cao C, Cerfolio RJ, Louie BE, Melfi F, Veronesi G, Razzak R (2019). Incidence, management, and outcomes of intraoperative catastrophes during robotic pulmonary resection. Ann Thorac Surg.

[12] Ueno H, Watanabe Y, Hirayama S, Hattori A, Imashimizu K, Takamochi K (2021). Intraoperative complications and troubles in robot-assisted anatomical pulmonary resection. Gen Thorac Cardiovasc Surg.

[13] Pfitzner J, Peacock MJ, Harris RJ (2001). Speed of collapse of the non-ventilated lung during single-lung ventilation for thoracoscopic surgery: the effect of transient increases in pleural pressure on the venting of gas from the non-ventilated lung. Anaesthesia.

[14] Ko R, McRae K, Darling G, Waddell TK, McGlade D, Cheung K (2009). The use of air in the inspired gas mixture during two-lung ventilation delays lung collapse during one-lung ventilation. Anesth Analg.

